# Antiobesity Effects of *Gentiana lutea* Extract on 3T3-L1 Preadipocytes and a High-Fat Diet-Induced Mouse Model

**DOI:** 10.3390/molecules25102453

**Published:** 2020-05-25

**Authors:** Eunkuk Park, Chang Gun Lee, Junho Kim, Subin Yeo, Ji Ae Kim, Chun Whan Choi, Seon-Yong Jeong

**Affiliations:** 1 Department of Medical Genetics, Graduate School of Medicine, Ajou University, Suwon 16499, Korea; jude0815@hotmail.com (E.P.); dangsunsang@naver.com (C.G.L.); 2 Department of Biomedical Sciences, Graduate School of Medicine, Ajou University, Suwon 16499, Korea; 3 Nine B Company, Daejeon 34121, Korea; kjh_nineb@naver.com (J.K.); snsnans@naver.com (S.Y.); ji.ae.kim@daum.net (J.A.K.); 4 Natural Product Research Team, Biocenter, Gyeonggido Business and Science Accelerator, Suwon 16229, Korea; cwchoi78@gmail.com

**Keywords:** *Gentiana lutea* L, obesity, 3T3-L1 cell, high fat diet, adipogenesis

## Abstract

Obesity is one of the most common metabolic diseases resulting in metabolic syndrome. In this study, we investigated the antiobesity effect of *Gentiana lutea* L. (GL) extract on 3T3-L1 preadipocytes and a high-fat-diet (HFD)-induced mouse model. For the induction of preadipocytes into adipocytes, 3T3-L1 cells were induced by treatment with 0.5 mM 3-isobutyl-1-methylxanthine, 1 mM dexamethasone, and 1 μg/mL insulin. Adipogenesis was assessed based on the messenger ribonucleic acid expression of adipogenic-inducing genes (adiponectin (*Adipoq*), CCAAT/enhancer-binding protein alpha (*Cebpa*), and glucose transporter type 4 (*Slc2a4*)) and lipid accumulation in the differentiated adipocytes was visualized by Oil Red O staining. In vivo, obese mice were induced with HFD and coadministered with 100 or 200 mg/kg/day of GL extract for 12 weeks. GL extract treatment inhibited adipocyte differentiation by downregulating the expression of adipogenic-related genes in 3T3-L1 cells. In the obese mouse model, GL extract prevented HFD-induced weight gain, fatty hepatocyte deposition, and adipocyte size by decreasing the secretion of leptin and insulin. In conclusion, GL extract shows antiobesity effects in vitro and in vivo, suggesting that this extract can be beneficial in the prevention of obesity.

## 1. Introduction

Obesity is the most common metabolic disease associated with several metabolic complications, including type 2 diabetes, hypertension, and cardiovascular disease [1]. Obesity is a disorder related to the imbalance of energy intake and expenditure, resulting in excess lipid accumulation in white adipose tissue [2].

Generally, long-term moderated lifestyle changes such as decreasing food intake and increasing physical activity have successfully reduced body weight in humans [3]. Thus, nutrition and exercise intervention are considered as effective strategies to prevent and manage obesity [4]. Currently, pharmacological drugs for the treatment of obesity can reduce appetite or decrease fat absorption [5]. However, the long-term use of pharmacotherapies for weight loss still has limitations with side effects.

Natural plants have been extensively used as alternative treatment of modern medicine in various diseases, as traditional natural medicines are appropriate for long-term treatment due to their lower adverse effects compared to pharmacological medications [2,3]. *Gentiana lutea* (GL) belongs to the Gentianaceae family, which comprises about 1600 species widely distributed in mountain areas of central and southern Europe [6,7]. The root of GL has traditionally been used as an herbal medicine to enhance digestion and gastrointestinal motility and improve liver function [8,9]. Additionally, the roots have several biological effects, such as antioxidant [10], radioprotective [11], and antimicrobial [12] activities. A previous study showed that the root of GL reduced total cholesterol in the blood of streptozotocin-induced diabetic rats [13]. However, the inhibitory effect of GL on obesity has not been investigated.

In the present study, we aimed to investigate the effect of GL on adipogenesis in preadipocyte 3T3-L1 cells and high fat diet (HFD)-induced obese mice.

## 2. Results and Discussion

### 2.1. Gentiana lutea (GL) Decreased Adipocyte Differentiation in 3T3-L1 Cells

Adipogenesis contributes to excess fat accumulation in adipocytes during the differentiation of preadipocytes. Mouse 3T3-L1 is the most commonly used preadipocyte cell line in the study of molecular and cellular mechanisms of adipogenesis [14]. Understanding the effect of adipogenesis has been considered a key issue for the development of antiobesity agents [2].

Firstly, we investigated the inhibitory effect of a GL extract on adipocyte differentiation in 3T3-L1 cells. Adipogenesis was assessed by the messenger ribonucleic acid (mRNA) expression of adipogenic-inducing genes, including adiponectin (*Adipoq*), CCAAT/enhancer-binding protein alpha (*Cebpa*), and glucose transporter type 4 (*Slc2a4*). Adiponectin is an adipocyte complement-related protein and it is produced and secreted by adipose cells that upregulate fatty acid synthase during lipid synthesis [15,16,17]. CCAAT/enhancer-binding protein alpha and peroxisome proliferator-activated receptor γ (PPARγ) are key transcription factors that cooperatively orchestrate adipocyte metabolism during 3T3-L1 adipocyte differentiation [18,19]. Glucose transporter type 4 is an insulin-regulated glucose transporter expressed primarily in adipose tissues that control adipose tissue mass [20]. These adipogenic-related genes play an important role in the control of lipid metabolism and adipogenesis.

**Figure 1:**
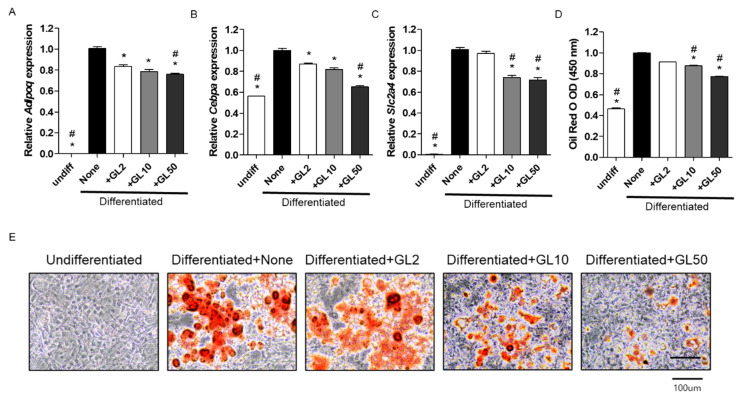
Effects of *Gentiana lutea* (GL) extract on the messenger ribonucleic acid (mRNA) expression of adipogenesis-inducing genes in 3T3-L1 preadipocyte cells. The cells were treated with 2, 10, or 50 μg/mL of GL extract (GL2, GL10, or GL50) for 8 days. The mRNA expression of (**A**) *Adipoq*, (**B**) *Cebpa*, and (**C**) *Slc2a4* was quantitatively analyzed by real-time polymerase chain reaction using gene-specific primers and subsequently normalized by the mRNA expression of *Gapdh*. The experiments were repeated at least three times using three independent samples. (**D**) Lipid accumulation in differentiated adipocytes was quantified based on the OD value of the cells at 450 nm. (**E**) Cells positively stained with Oil Red O were visualized under a microscope. At least three different images were captured for each sample. * *p* < 0.05 vs. none, # *p* < 0.05 vs. GL2 (Tukey’s honest significant difference post hoc test, analysis of variance). Abbreviations: Undiff, undifferentiated; None, non-treated.

Regarding adipocyte differentiation, 3T3-L1 preadipocyte cells were induced by adding 0.5 mM 3-isobutyl-1-methylxanthine, 1 mM dexamethasone, and 1 μg/mL insulin (MDI) [14]. Lipid-rich vacuoles apparent in the differentiated adipocytes were histologically stained with Oil Red O [21]. Oil Red O staining as a reliable indicator of adipogenesis has been used for the qualitative measurement of adipocyte differentiation in 3T3-L1 preadipocyte cells [14]. Treatment of GL extracts (2, 10, and 50 μg/mL) did not affect the cell proliferation during the differentiation period of 8 days in 3T3-L1 preadipocytes (data not shown). The mRNA expression levels of *Adipoq, Cebpa*, and *Slc2a4* were enhanced by inducing adipocyte differentiation (Figure 1A–C). However, GL treatment significantly inhibited mRNA expression of adipogenesis-related genes (*Adipoq, Cebpa*, and *Slc2a4*) (Figure 1A–C). The number of Oil Red O staining was increased by inducing adipocyte differentiation but was reduced upon GL treatment (Figure 1D,E). These results suggested that GL treatment prevented adipocyte differentiation by downregulating the expression of adipogenic-inducing genes in 3T3-L1 preadipocytes.

### 2.2. GL Reduced Obesity-Related Phenotypes in High-Fat Diet-Induced Mice

**Figure 2:**
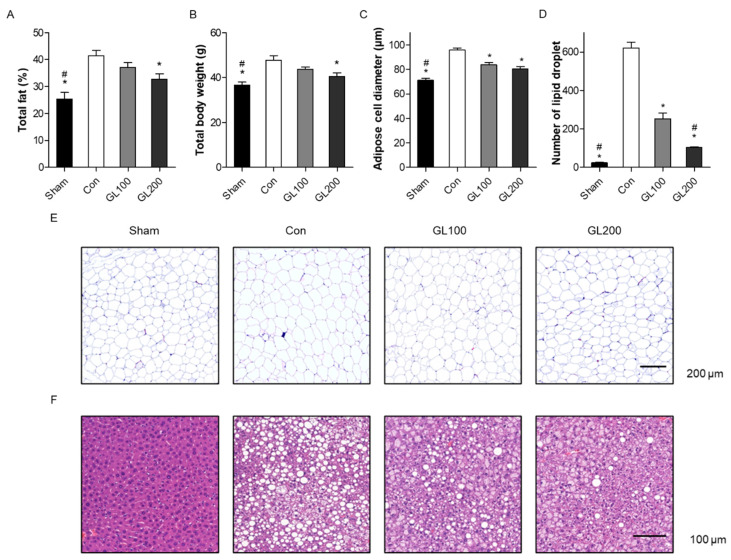
Antiobesity effects of *Gentiana lutea* (GL) extract on high fat diet (HFD)-induced obese mice. After 12 weeks of experiment, (**A**) the total fat% and (**B**) total body weight of mice were measured in normal diet (Sham), HFD (Control (Con)), and HFD mice with the administration of 100 (GL100) or 200 (GL200) mg/kg/day of GL extract. The total fat% and total body weight of all mice were measured using a PIXImus small animal densitometer and an electronic scale, respectively. (**C**) Hematoxylin and eosin (H&E)-stained adipose cell diameters were individually measured using the CaseViewer program. (**D**) The number of lipid deposition in H&E-stained liver was calculated in at least five images in each group. * *p* < 0.05 vs. Con. # *p* < 0.05 vs. GL100. (Tukey’s honest significant difference post hoc test, analysis of variance). H&E-stained images of the (**E**) abdominal visceral fat and (**F**) liver tissues. Six animals in each group were used for this experiment.

Based on results from the in vitro study, we further examined the antiadipogenic effects of GL extract in the HFD-induced obesity animal model. Four-week-old male C57BL/6J mice were fed a 60% fat diet and normal diet (Sham) and divided into the following four groups: (1) Sham (normal diet), (2) Control (60% fat diet), (3) GL 100 (60% fat diet + 100 mg/kg/day of GL extract), and (4) GL 200 (60% fat diet + 200 mg/kg/day of GL extract). HFD animals are well-known models of human obesity characterized by an increase in the total body weight and total fat% [22]. During the experimental period, animals appeared healthy with no pathological signs or abnormalities, and food intake did not differ between the HFD and GL extract-treated groups (data not shown). All four groups had similar body weights at the beginning of the study. At the end of the experiment, the total fat% and total body weight were measured using a PIXImus bone densitometer. The total fat% was analyzed as the total body fat divided by total body weight. HFD for 12 weeks significantly increased the total fat% and total body weight. However, treatment with 200 mg/kg/day of GL extract inhibited HFD-induced total fat% and total body weight compared to the control group (Figure 2A,B).

Subsequently, the morphology of the liver and abdominal visceral fat tissues of all mice were stained using hematoxylin and eosin (H&E) staining, and adipocyte diameters were measured using the CaseViewer program (3DHISTECH Ltd.). HFD mice presented with severe cytoplasmic vacuoles and a high steatosis with hepatocyte swelling in liver tissues and enlarged adipocytes in abdominal visceral fat tissue. However, GL treatment prevented any increase in the diameter and size of the adipocytes (Figure 2C,D) and reduced lipid deposition in hepatocytes (Figure 2D,E). These results suggested an antiobesity effect of GL extract in the HFD-induced animal model.

### 2.3. GL Inhibited High-Fat Diet-Induced Serum Levels of Obesity-Related Hormones

Next, we confirmed the antiobesity effects of GL extract on obesity-associated hormones (leptin and insulin). The blood samples were collected from the left ventricle of the anesthetized mice at the last day of treatment, and serum levels of leptin and insulin were measured using the multiplex assays analyzed with Luminex. Leptin, an adipocyte-derived hormone, is closely associated with the body mass index and adipose tissue mass [23]. A previous study reported that increased body weight enhances the levels of adipocyte-derived leptin during HFD, whereas inhibition of adipocyte differentiation results in decreased serum concentration of leptin [24]. Insulin is one of the critical regulators of adipogenesis that increases the uptake of fatty acids and glucose transport [25,26]. Additionally, insulin and leptin hormones are associated with obesity and positively co-associated with the body weight and fat mass [27,28].

**Figure 3:**
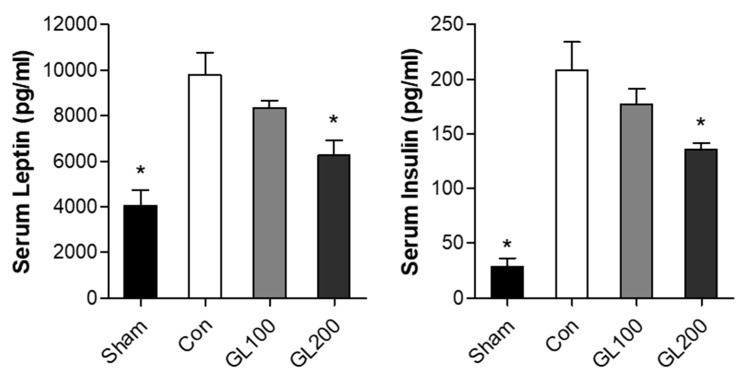
Effects of *Gentiana lutea* extract on serum levels of adipogenic makers (leptin and insulin). After 12 weeks of administration, the serum levels of leptin and insulin were measured using multiplex assays analyzed with Luminex. *p < 0.05 vs. Con. (Tukey’s honest significant difference post hoc test, analysis of variance). The experiment was performed using blood samples (*n* = 5) and was repeated at least three times.

A previous study demonstrated that a linear correlation was observed between weight gain and the leptin and insulin levels in humans [29]. Similarly, our results showed that HFD-induced mice increased leptin and insulin levels (Figure 3). However, the serum concentration of leptin and insulin were reduced by the treatment of 200 mg/kg/day of GL extract. These results indicate that the GL extract inhibited HFD-induced weight gain and decreased the secretion of leptin and insulin in the obese mouse model.

### 2.4. Extraction and Fractionation of Gentiana lutea Linne Roots

**Figure 4:**
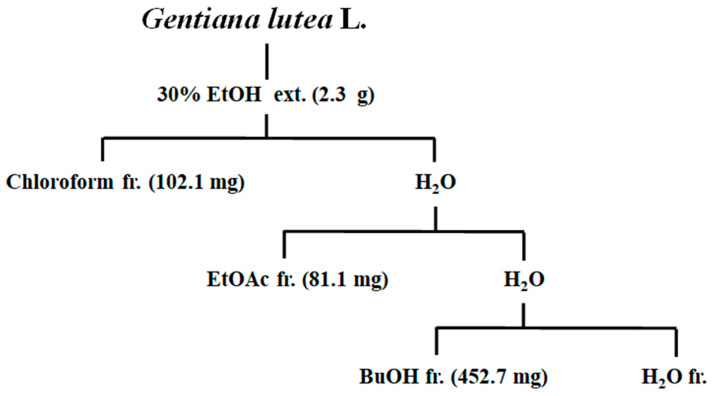
Extraction and fractionation (fr.) of major components from *Gentiana lutea* L.

To isolate the main constituents of the roots of *Gentiana lutea* L. extract, GL (50 g) plants were extracted for 24 h at room temperature with 30% ethanol (2 × 0.5 L). After removing the ethanol under vacuum, the aqueous solution was filtered through filter paper. The filtrate was concentrated and the crude extract (2.3 g) was dissolved in 25 mL of H_2_O to form a suspension that was successively partitioned with chloroform, EtOAc, and BuOH to give chloroform (102.1 mg), EtOAc (81.1 mg), and BuOH (452.7 mg) extracts, respectively. The extraction and fractionation methods are shown in Figure 4. The fractionation of the 30% ethanol extract from roots of *Gentiana lutea* L. and the evaluation of the anti-adipogenic effect of the 30% ethanol extract, chloroform, EtOAc, BuOH, and water fractions were conducted following the above assay. The BuOH fractions had potent anti-adipogenic activities. From this activity, guided fractionations were determined according to the following procedure.

### 2.5. Isolation and Determination of Compounds **1** and **2** from the BuOH Fraction

**Figure 5:**
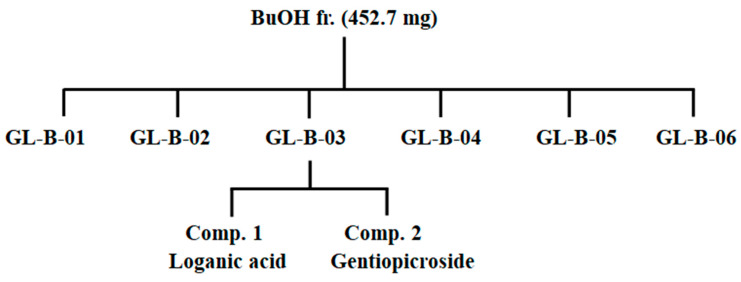
Isolation of compounds from BuOH fractions of *Gentiana lutea* L.

The BuOH extract was subjected to column chromatography using octadecylsilyl (ODS) gel, and eluted with MeOH/H_2_O (1/4, *v/v*) to give six fractions (GL-B-01 to 06). Fraction GL-B-03 (147.6 mg) was subjected to RP-18 column chromatography and eluted with acetonitrile (CAN) in H_2_O (0.05% trifluoroacetic acid (TFA)) in a step-gradient manner (10% to 40%) to produce compound **1** and **2** (Figure 5). The structures of Compounds **1** and **2** were elucidated by chemical evidence on the basis of NMR spectroscopic and MS data, and as well as by comparison with those reported [30,31].

### 2.6. Physicochemical and Spectroscopic Properties of Isolated Compounds

Compound **1:** ^1^H-NMR (400 MHz, CD_3_OD): δ 5.22 (1H, d, *J* = 4.1 Hz, H-1), 7.29 (1H, s, H-3), 3.05 (1H, q, *J* = 8.0 Hz, H-5), 1.62 (1H, dd, *J* = 13.7, 5.9 Hz, H-6a), 2.18 (1H, dd, *J* = 13.7, 7.8 Hz, H-6b), 3.99 (1H, t, *J* = 5.1 Hz, H-7), 1.82 (1H, m, H-8), 1.97 (1H, td, *J* = 9.0, 4.2 Hz, H-9), 1.04 (3H, d, *J* = 6.8 Hz, H-10), 4.60 (1H, d, *J* = 7.9 Hz, H-1′), 3.15 (1H, d, *J* = 8.5 Hz, H-2′), 3.32 (1H, m, H-3′), 3.21 (1H, m, H-4′), 3.33 (1H, m, H-5′), 3.61 (1H, dd, *J* = 11.9, 5.4 Hz, H-6′a), 3.84 (1H, d, *J* = 11.9 Hz, H-6′b).^13^C-NMR (100 Hz, CD_3_OD): δ 97.5 (C-1), 151.3 (C-3), 115,0 (C-4), 32.2 (C-5), 42.7 (C-6), 74.7 (C-7), 42.1 (C-8), 46.6 (C-9), 13.4 (C-10), 171.8 (C-11), 100.0 (C-1′), 75.1 (C-2′), 78.0 (C-3′), 71.6 (C-4′), 78.3 (C-5′), 62.7 (C-6′).

Compound **2:** ^1^H-NMR (700 MHz, DMSO-*d_6_*): δ 7.41 (1H, d, *J* = 0.02 Hz, H-3), 5.71 (1H, m, H-8), 5.64 (1H, m, H-6), 5.59 (1H, d, *J* = 3.5 Hz, H-1), 5.21 (1H, dt, *J* = 1.4, 9.45 Hz, H-10a), 5.19 (1H, m,H-10b), 5.04 (1H, m, H-7a), 4.98 (1H, m, H-7b), 4.55 (1H, s, H-2′),4.48 (1H, d, J = 7.7 Hz, H-1′), 3.68 (1H, dd, J = 2.8, 11.55 Hz, H-6a′), 3.42 (1H, m, H-6b′), 3.29-3.32 (1H, m, H-9), 3.12-3.16 (1H, m, H-5′), 2.99–3.02 (1H, m, H-3′), 2.92–2.95 (1H, m,H-4′).^13^C-NMR (DMSO-*d_6_*, 125 MHz): δ 162.8 (C-11), 148.8 (C-3), 134.0 (C-8), 125.0(C-5), 118.0 (C-10), 116.2 (C-6), 103.3 (C-4), 98.8 (C-1), 96.5 (C-1′), 77.4 (C-5′),76.7 (C-3′), 72.8 (C-2′), 70.0 (C-4′), 69.2 (C-7), 61.1 (C-6′), 44.4 (C-9).

**Figure 6:**
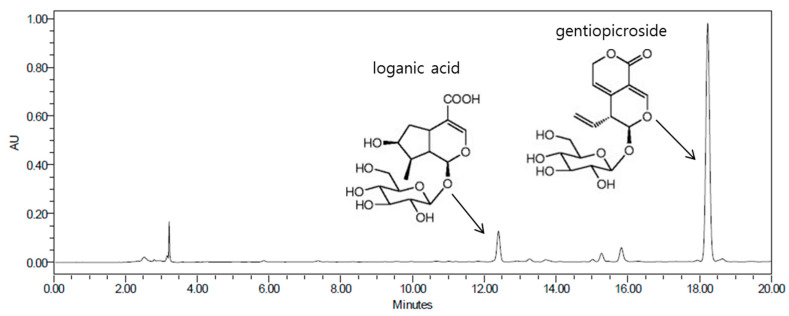
High-performance liquid chromatography (HPLC) analysis of *Gentiana lutea* L. *extract*.

High-performance liquid chromatography (HPLC) chromatogram of the GL extract showed that the most abundant constituents were loganic acid and gentiopicroside. The proposed chemical structures of the two constituents (loganic acid and gentiopicroside) are shown in Figure 6. Previous studies have suggested that loganic acid and gentiopicroside decrease adipocyte differentiation of 3T3-L1 preadipocytes and inhibit body weight gain in an ovariectomy-induced obesity mouse model [32,33]. These results support the hypothesis that GL extract containing loganic acid and gentiopicroside as major components has enhanced the antiobesity effects on 3T3-L1 preadipocytes and the HFD-induced mouse model. A limitation of this study is that it is still not known if the concentrations of GL in cell culture experiments are physiological and it is unclear if one or multiple components of GL are responsible for the observed effects. Because this study was conducted only in male mice, it is uncertain whether the anti-adipogenic effect of GL extract is gender-specific. In addition, detailed anti-obesity effects of GL are required for further study with other variables including energy expenditure from basal metabolic rate or activity level.

## 3. Materials and Methods Section

### 3.1. GL Extract and Cell Culture

The *Gentiana lutea* was purchased from the TCM market in Seoul, Korea, in September 2017. Voucher specimens (GL0180), authenticated by Dr. Chun Whan Choi, were deposited at the herbarium of Bio-center, Gyeonggi Business & Science & Accelerator, Suwon, South Korea. ^1^H and ^13^C NMR experiments were performed on a Bruker Ascend 700 MHz spectrometer (Bruker BioSpin AG, Faellanden, Switzerland) with tetramethylsilane (TMS). Liquid Chromatography Electrospray Ionization Tandem Mass Spectrometry (LC-ESI-MS) was performed on a Triple TOF 5600+ instrument (AB SCIX, Redwood City, CA, USA) and high-resolution electrospray ionisation mass spectrometry (HRESI-MS) on an LTQ Orbitrap XL instrument (Thermo, Waltham, MA, USA). Thin Layer Chromatography (TLC) was conducted on Silica gel 60 F254 (Merck, Darmstadt, Germany) and Silica gel 60 RP-18 F254S (Merck, Darmstadt, Germany) plates. Column chromatography (CC) was performed using Silica gel 60 (70~230 mesh, Merck, Darmstadt, Germany), ODS-A (12 nm S-7 μm, YMC GEL, Tokyo, Japan), and Preparative HPLC was performed on LC-8A (Shimadzu, Kyoto, Japan).

Mouse 3T3-L1 preadipocytes were incubated in Dulbecco’s modified Eagle’s medium supplemented with streptomycin (100 µg/mL), penicillin (100 U/mL), and 10% fetal bovine serum. Regarding the differentiation of adipocytes, preadipocytes were induced by adding 1 µg/mL insulin, 1 mM dexamethasone, and 0.5 mM 3-isobutyl-1-methylxanthine and co-treated with 2, 10, or 50 µg/mL of GL for 3 days. The induction medium was changed with 1 µg/mL insulin for another 5 days, and a fresh medium was changed every 3 days. During incubation, cells were cultured under a controlled humidified atmosphere at 37 °C and 5% CO_2_ atmosphere.

### 3.2. Water-Soluble Tetrazolium Salt Assay

Preadipocyte cells were incubated in a 96-well plate overnight and co-treated with three different concentrations of GL extract (2, 10, and 50 µg/mL) in the medium for 8 days. The water-soluble tetrazolium (WST) salt assay was analyzed using an EZ-Cytox Cell Viability Assay Kit (Daeil; Seoul, Korea). WST solution containing 20 µL and 5 mg/mL phosphate-buffered saline was treated in each well and kept for another 4 h. The absorbance of cell viability was read at 450 and 655 nm using a microplate reader (BioTek, Winooski, VT, USA).

### 3.3. Oil Red O Staining of Adipocytes

After the differentiation of preadipocyte cells, neutral lipids were measured using Oil Red O staining. Lipid accumulation of differentiated adipocytes was fixed with 4% paraformaldehyde for 15 min. After removing the media, cells were washed with PBS three times and stained with Oil Red O dye for 2 h. The number of Oil Red O-stained cells was calculated in at least three images for each group. The lipid accumulation was visualized under a microscope at 100X magnification. For quantification of lipid accumulation, the Oil Red O-positive cells were extracted using 100% isopropanol for 10 min. The absorbance of the extracted dye was analyzed at a wavelength of 450 nM (BIO-RAD; Hercules, CA, USA).

### 3.4. Quantitative Reverse Transcription Polymerase Chain Reaction

The total RNA of 3T3-L1 cells was extracted using a TRIzol reagent (Invitrogen, Carlsbad, CA, USA) according to the manufacturer’s instructions (Beckman Coulter, Brea, CA, USA). The extracted total RNA was subsequently reverse transcribed using a Synthesis Kit of RevertAid™ H Minus First Strand cDNA (Fermentas, Inc., Vilnius, Lithuania) with random hexamer and oligo(dT)_15–18_ primers. A total volume of 25 µL containing 150 ng of cDNA within a SYBR Green I qPCR kit (Takara, Shiga, Japan) was analyzed by real-time polymerase chain reaction using an ABI Prism 7000 Sequence Detection System (Applied Biosystems, Foster City, CA, USA). All PCR amplifications were conducted in 25 μL PCR mixtures containing 150 ng of cDNA, using a SYBR Green I qPCR kit (TaKaRa, Shiga, Japan), according to the manufacturer′s recommendations. The cycling conditions included 40 cycles of amplification. The specific primers used for adipogenesis-associated genes were: for mouse *Adipoq,* 5′-TGT TCC TCT TAA TCC TGC CCA-3′ and 5′-CCA ACC TGC ACA AGT TCC CTT-3′; for mouse *Cepba,* 5′-GCG GGA ACG CAA CAT C-3′ and 5′-GTC ACT GGT CAA CTC CAG CAC-3′; for mouse *Slc2a4*, 5′-ACA CTG GTC CTA GCT GTA TTC T-3′ and 5′-CCA GCC ACG TTG CAT TGT A-3′; and for mouse *Gapdh*; 5′-TGA CCA CAG TCC ATG CCA TC-3′ and 5′-GAC GGA CAC ATT GGG GGT AG-3′. The expression levels of related genes were normalized to that of *Gapdh* and expressed in terms of fold-change compared to the control. The experiment was repeated at least three times using three independent samples.

### 3.5. In Vivo Experiments in Obesity Model Mice

Four-week-old male C57BL/6J mice were fed a 60% fat diet (HFD, *n* = 24) and normal diet (Sham, *n* = 8) purchased from ORIENT BIO Inc. (Seongnam-si, Korea). Mice were fed with a normal diet or HFD (60% fat) and tap water (15 mL/day) and treated with (1) Sham (normal diet), (2) Control (60% fat diet), (3) GL 100 (60% fat diet + 100 mg/kg/day of GL extract), and (4) GL 200 (60% fat diet + 200 mg/kg/day of GL extract). The combination of GL was orally administered at two different concentrations (100 and 200 mg/kg/day, *n* = 5 in each group). All mice were maintained in clear plastic cages under a certain temperature (23 ± 2 °C), humidity (55 ± 5%), and illumination (12 h light/dark cycle). All experiments were conducted according to the institutional guidelines from the Committee of the Ajou University School of Medicine, Suwon, South Korea (AMC-133).

### 3.6. Preparation of Tissue Samples and Histology

Liver and abdominal fat tissues were removed, fixed in 4% paraformaldehyde, and embedded in paraffin. The embedded tissues were sectioned (3 μM) in the transverse plane. The tissue sections were deparaffinized using xylene, rehydrated with ethanol solutions of different concentrations, and stained with H&E. The histological changes in the stained tissues sections were evaluated under a microscope. At least three images were taken for each sample.

### 3.7. Blood Sampling and Serum Leptin and Insulin

Animals were anesthetized with tiletamine/zolazepam (Zoletil; Virbac Laboratories, Carros, France), and blood samples from the left ventricle were collected using heparinized syringes. Serum samples were separated by centrifugation at 1200× *g* at 4 °C for 15 min and measured for adipogenic makers (leptin and insulin) using customized multiplex assays analyzed with Luminex (Merck Millipore, Burlington, MA, USA) following the manufacturer’s instructions. The experiment was repeated at least three times using at least 5 blood samples.

### 3.8. Statistical Analysis

A statistical software package PASW Statistics version 17 (Statistical Package for the Social Sciences, Inc., Chicago, IL, USA) was used in performing the statistical tests. A one-way analysis of variance was used for multiple groups and compared using Tukey’s honest significant difference post hoc test for the correction. A value of *p* < 0.05 was considered significant. The results were expressed as mean ± standard error of the mean.

## 4. Conclusions

We described the antiadipogenic effects of GL extract on preadipocyte 3T3-L1 cells and HFD-induced obese mice. GL extract reduced the expression of adipogenic-related genes in differentiated 3T3-L1 cells, resulting in the inhibition of adipocyte differentiation. HFD-induced weight gain and obesity-associated hormones in mice were prevented by the treatment of GL extract. This study suggests that GL extract may be a potential therapeutic strategy for the prevention of obesity.
